# P02 Evaluating antibiotic prescribing in pancreatitis: a single-centre retrospective case series

**DOI:** 10.1093/jacamr/dlad143.006

**Published:** 2024-01-03

**Authors:** Miriam Beattie, Daniel Hearsey, Neil Powell

**Affiliations:** Royal Cornwall Hospital, Truro, Cornwall, UK; Royal Cornwall Hospital, Truro, Cornwall, UK; Royal Cornwall Hospital, Truro, Cornwall, UK

## Abstract

**Background:**

Procalcitonin (PCT) has been shown to safely reduce antibiotic use in respiratory tract infections but there is little evidence for other potentially infected body systems. A recent randomized controlled trial (RCT) demonstrated the safety and efficacy of PCT in reducing antibiotic use in patients with pancreatitis.

**Objectives:**

We aimed to review antibiotic prescribing in patients with pancreatitis and to estimate the potential impact of PCT on rates of antibiotic prescribing at the Royal Cornwall Hospital Trust (RCHT).

**Methods:**

Patient care episodes for pancreatitis between June 2022 and May 2023 were identified using pancreatitis ICD-10 codes. An audit tool was developed using the inclusion criteria used in the RCT. Electronic notes systems were used to collect the following: patient demographics, whether diagnostic criteria for pancreatitis were met, clinical specialty, pancreatitis severity, presence of necrosis, indications for antibiotics and course lengths.

**Results:**

121 patient care episodes were identified, of which 76 (63%) were analysed. Of these, 61 (80.3%) met the diagnostic criteria for pancreatitis and analysed; median age 57 (IQR 26), 28 (45.9%) female, 51 (83.6%) managed under surgeons and 10 (16.4%) under medicine. 15 (19.7%) patient episodes were excluded: 14 did not meet diagnostic criteria for pancreatitis and 1 on long term immunosuppression. 30 (49.2%) patients with a clinical diagnosis of pancreatitis were prescribed antibiotics; 5 (16.7%) for pancreatitis and 16 (53.3%) for extra-pancreatic indications: respiratory tract 3 (18.8%), cholecystitis 7 (43.8%), suspected cholangitis 1 (6.3%), other intra-abdominal infection 5 (31.3%). The indication for antibiotics was unclear in 9 episodes (30.0%). Of those prescribed antibiotics for pancreatitis, 4 (80%) were appropriately prescribed for suspected infected necrotic pancreatitis (patients with pancreatic or extra-pancreatic necrosis who deteriorate (physiologically or blood results) or fail to improve after 7-10 days), 1 (20%) was inappropriate. The overall rate of antibiotic prescribing in the PROCAP study were 31% in the PCT group and 47% in the usual care group (non-PCT); the rate of antibiotic prescribing in the usual care group was found to be comparable to our data (47.0% verses 49.2%). However, the majority of antibiotic prescriptions in our cohort were for extra-pancreatic infections, not pancreatitis. It is not possible to determine the proportion of antibiotics prescribed for pancreatitis in the PROCAP study and therefore we are unable to compare our findings.

**Conclusions:**

Rates of antibiotic prescribing for patients at RCHT with pancreatitis are comparable to those in the PROCAP non-PCT guided treatment group which might suggest that adoption of PCT in our hospital could reduce antibiotic prescribing, as per the findings of the PROCAP study. Rates of antibiotic prescribing for pancreatitis at RCHT are low, with the majority of prescribing for extra-pancreatic indications, although the indication was unclear in approximately one third of episodes. The PROCAP study does not report rates of prescribing for pancreatitis which prevents estimating the impact of PCT on antimicrobial prescribing for pancreatitis at RCHT. Whether PCT has a role in reducing antimicrobial prescribing for patients with pancreatitis at RCHT is unclear.

